# Understanding the Relationship between Type 2 Diabetes Mellitus and Falls in Older Adults: A Prospective Cohort Study

**DOI:** 10.1371/journal.pone.0067055

**Published:** 2013-06-25

**Authors:** Tine Roman de Mettelinge, Dirk Cambier, Patrick Calders, Nele Van Den Noortgate, Kim Delbaere

**Affiliations:** 1 Department of Rehabilitation Sciences and Physiotherapy, Faculty of Medicine and Health Sciences, Ghent University, Ghent, Belgium; 2 Department of Internal Medicine, Division of Geriatrics and Gerontology, Ghent University Hospital, Ghent Belgium; 3 Falls and Balance Research Group, Neuroscience Research Australia, Randwick, Sydney, Australia; Cardiff University, United Kingdom

## Abstract

**Background:**

Older adults with type 2 Diabetes Mellitus are at increased risk of falling. The current study aims to identify risk factors that mediate the relationship between diabetes and falls.

**Methods:**

199 older adults (104 with diabetes and 95 healthy controls) underwent a medical screening. Gait (GAITRite®), balance (AccuGait® force plate), grip strength (Jamar®), and cognitive status (Mini-Mental State Examination and Clock Drawing Test) were assessed. Falls were prospectively recorded during a 12-month follow-up period using monthly calendars.

**Results:**

Compared to controls, diabetes participants scored worse on all physical and cognitive measures. Sixty-four participants (42 diabetes vs. 22 controls) reported at least one injurious fall or two non-injurious falls (“fallers”). Univariate logistic regression identified diabetes as a risk factor for future falls (Odds Ratio 2.25, 95%CI 1.21–4.15, *p* = 0.010). Stepwise multiple regressions defined diabetes and poor balance as independent risk factors for falling. Taking more medications, slower walking speed, shorter stride length and poor cognitive performance were mediators that reduced the Odds Ratio of the relationship between diabetes and faller status relationship the most followed by reduced grip strength and increased stride length variability.

**Conclusions:**

Diabetes is a major risk factor for falling, even after controlling for poor balance. Taking more medications, poorer walking performance and reduced cognitive functioning were mediators of the relationship between diabetes and falls. Tailored preventive programs including systematic medication reviews, specific balance exercises and cognitive training might be beneficial in reducing fall risk in older adults suffering from diabetes.

## Introduction

Diabetes Mellitus and falls are common in the older population and can therefore be considered ‘geriatric giants’. They both pose major threats to an older person's quality of life. According to the World Health Organization, diabetes globally affects approximately 347 million people and diabetes deaths will double between 2005 and 2030 [1]. Each year, approximately one in three community-dwelling older adults aged 65 or over suffers one or more falls [2]. Older women with diabetes are 1.6 times more likely to have fallen in the previous year and twice as likely to have had injurious falls [3]. Diabetes Mellitus has been identified as a risk factor for falls [4], [5] and fall-related injuries and fractures [6] in a number of prospective studies.

Poor balance has been determined as a major risk factor for falls in older adults [7]. Many diabetes-related complications, such as peripheral neuropathies [8], cerebrovascular accidents [9], sarcopenia [10], poor low-contrast visual acuity and poor depth perception [11] have also been associated with reduced balance performance [7]. Other complications from diabetes, such as urinary incontinence [12], dementia [13], mild cognitive impairment [14] and depressive symptoms [15], have been identified as risk factors for falls in older adults without diabetes. However, due to a lack of comprehensive prospective studies focusing on fall risk detection in older adults with diabetes, it is unclear whether these factors mediate the relationship between falls and diabetes. The aims of this study are therefore (i) to establish distinguishing factors between older adults with and without diabetes on a range of established fall risk factors, (ii) to document fall rates and determine fall risk factors in a matched cohort of older adults with and without diabetes, and (iii) to identify mediating risk factors of falling that explain the relationship between diabetes and falls in older adults. This will assist in designing tailored fall prevention programs in this population.

## Materials and Methods

### Ethics Statement

The Ethical Committee of the Ghent University Hospital gave approval to this study and all participants signed an informed consent.

### Participants

199 older adults were enrolled in this study. The general practitioner or medical specialist of each participant confirmed the presence or absence of type 2 Diabetes Mellitus. Inclusion criteria were: (i) aged 60 years and above, (ii) living in the community or residential aged care setting, (iii) able to understand instructions, (iv) able to walk independently with or without walking aids, (v) absence of stroke, Parkinson's disease or other major neurological conditions, and (vi) absence of musculoskeletal disorders impeding them to walk unaided for 10 m (e.g. amputations, major rheumatic conditions in the lower extremity). Seventy-two (69.2%) older adults with diabetes and 43(45.3%) healthy controls were recruited from residential aged care settings. Eleven (10.6%) community-dwelling older adults with diabetes and 52(54.7%) community-dwelling healthy controls were recruited through online advertising, flyer distribution and by word of mouth. Another 21(20.2%) older adults with diabetes were recruited from the Endocrinology Clinic at the Ghent University Hospital, Belgium.

### Personal and Medical History

Socio-demographic data and medical history were recorded by means of a self-report questionnaire. Participants were asked about previous falls, fear of falling (yes/no), number of medications and pathological conditions potentially interfering with fall risk such as depression or urinary incontinence. Peripheral nerve function was assessed by determination of the Vibration Perception Threshold, which has proven reliability and validity towards assessment of neural dysfunction in people with diabetes [16]. It was determined using a Bio-Thesiometer® (Bio Medical Instrument co, Ohio, USA) by three measurements on four distinct points (medial malleolus and big toe on both feet). For each location the mean of three values was calculated.

### Physical Measurements

#### Muscle Strength

Grip strength (kg) of the dominant hand was recorded using the Jamar® dynamometer (Sammons Preston Rolyan Inc., Bolingbrook, IL) while seated in an armless chair with shoulders adducted and neutrally rotated and elbow flexed at 90°, forearms in neutral position and wrist between 0 and 30° of dorsiflexion [17]. Participants were instructed to squeeze the handle as hard as possible [18]. The maximal grip score of three trials was retained.

#### Gait

Gait velocity (cm/s), stride length (cm) and stride length variability (%) were captured by the portable electronic GAITRite® walkway system (8.3 m×0.89 m; CIR Systems Inc., Havertown, PA, USA) with proven validity [19]. Stride length variability was calculated as the ratio of the standard deviation to the mean. Participants were asked to walk at a self-selected normal walking speed wearing comfortable footwear with a low and wide heel and a thin, grooved and moderately hard sole. Thirty-four percent (n=68) used their usual walking aid such as crutches, walkers or canes. Participants were instructed to start walking two meters before the GAITRite® mat and keep walking for two meters beyond the mat to minimize acceleration and deceleration effects.

#### Balance

Limits of stability (LOS) were determined by use of a force plate (AMTI® AccuGait, Advanced Mechanical Technology Inc., Watertown, MA, USA). Sampling rate was set at 50Hz and data were filtered with a cut-off frequency of 5Hz by a 4-th order low-pass Butterworth filter. Participants were instructed to position their feet shoulder-with apart and lean forward, backward, to the left and to the right as far as possible without moving their feet. LOS were expressed as maximal medio-lateral and antero-posterior displacement (cm) of the Center of Pressure (COP) and sway area (cm^2^). The sway area is the surface of an ellipse wherein 95% of the COP samples are predicted to be enclosed (95% confidence ellipse).

### Cognitive Measurements

The Mini-Mental State Examination (MMSE) was used as a general cognitive screening instrument [20]. The Clock Drawing Test (CDT) was done to estimate executive functioning. Four items as proposed by Thalmann et al. were selected: item 2(12 numbers are present), item 5(number ‘12’ correctly placed), item 25(hands have correct proportions) and item 34(participant reads time correctly) [21]. A validated algorithm to combine results from the MMSE and the CDT was used to estimate executive functioning [21]. MMSE score of 27 or more was coded as 3, and MMSE score of 26 or less was coded zero. The four CDT items were coded as 0 or 1 for items 2, 25 and 34; and as 0 or 3 for item 5. These recoded scores of the MMSE and the CDT were then combined to a single score (MMSE-CDT) with a maximum of 9, representing a good cognitive function. A cut-off score of less than 7 on the MMSE-CDT was used to classify participants as having reduced cognitive functioning.

### Falls Follow-Up

After baseline measurements falls were monitored during 12 months using monthly fall calendars. A fall was defined as “an unexpected event in which the person comes to rest on the ground, floor, or lower level” [22]. If a fall occurred, participants were telephoned and asked about the circumstances and fall injuries such as bruises, lacerations or fractures. Participants who reported multiple (>1) falls or at least one fall with injury were categorized as “fallers” whereas participants who experienced no fall or one non-injurious fall were considered “nonfallers” [23]. Two participants were lost to follow-up (1 control withdrew, 1 diabetes died) and were not included in statistical analyses.

### Statistical analyses

Univariate and multivariate logistic regression models were applied to investigate the association between diabetes and falls, and between covariates (demographic, medical, physical, cognitive) and falls. Covariates with a univariate statistical significance of *p*≤.1 were first entered in separate logistic regression models to determine how much they reduced the diabetes-falls Odds Ratio (OR). Covariates that mediated this relationship were then combined in a final logistic regression model. Marker variables such as “previous falls” were not selected as possible predictors in multivariate models as such marker variables often cancel out the impact of other risk factors and are therefore not helpful in assisting our understanding of why falls occur [24]. Independent Samples t tests (continuous variables) and Chi Square tests (categorical variables) were performed to compare healthy controls and older adults with diabetes. Data were analyzed using SPSS.20 for Windows (SPSS, Inc., Chicago, IL). For reasons of voluntary withdrawal, illness and absence at the time of the test procedure eight participants (2 controls and 6 with diabetes) did not complete gait analysis, fifteen (2 controls and 13 with diabetes) had no LOS data and four (4 with diabetes) performed no grip strength measurement.

## Results

Mean age of the 199 participants was 76.9 ± 9.4 (range 60–94) and 126 (63.3%) were female. Participants with diabetes (n=104) were older than controls (n=95), with a mean age of 78.4(SD 8.7) and 75.1(SD 9.9) respectively (Table 1). They took 2.1(SD 0.7) anti-diabetic agents on average and 44.1% were insulin-dependent. Fifty-six (28.4%) participants reported multiple falls during the 12 months follow up, eight (4.1%) reported one injurious fall, thirty-two (16.2%) reported 1 non-injurious fall and 101(51.3%) reported no falls. Forty-two (40.8%) older adults with diabetes reported one single injurious fall or multiple falls compared to 22(23.4%) healthy controls.

**Table 1:** Comparison of the Univariate Risk Factors between Healthy Controls and Diabetes Patients (n=199).

Risk Factor	Controls (n=95)	Diabetes (n=104)	*p* Value	Nonfallers (n=133)	Fallers (n= 64)	Odds Ratio (95% CI)
**Demographic/Medical**						
Age *(years)*	75.14 ± 9.86	78.41 ± 8.73	.014	75.8 ± 9.4	78.8 ± 9.3	1.38 (1.01–1.87)*
Female	62 (65.3)	64 (61.5)	.659	80 (60.2)	44 (68.8)	1.46 (0.77–2.74)
Body Mass Index *(kg/m^2^)*	27.46 ± 4.19	29.39 ± 5.73	.007	28.2 ± 4.9	29.1 ± 5.5	1.20 (0.89–1.60)
Community-dwelling	52 (54.7)	32 (30.8)	.001	62 (46.6)	22 (34.4)	1.67 (0.90–3.09)
Walking Aids	22 (23.2)	46 (44.2)	.003	39 (29.3)	29 (45.3)	2.00 (1.08–3.70)*
Number of medications	3.9 ± 3.1	9.0 ± 2.9	<.001	6.1 ± 3.9	7.6 ± 3.8	1.46 (1.07–2.00)*
Diabetes Mellitus	0 (0.0)	100 (100)	-	61 (45.9)	42 (65.6)	2.25 (1.21–4.18)**
Depression	12 (12.6)	22 (21.2)	.133	20 (15.0)	14 (21.9)	1.58 (0.74–3.38)
Urinary Incontinence	18 (18.9)	22 (21.2)	.726	18 (13.5)	21 (32.8)	3.12 (1.52–6.41)**
Fear of Falling	46 (48.4)	69 (66.3)	.014	66 (49.6)	48 (75.0)	3.05 (1.57–5.89)**
Previous Falls	24 (26.1)	53 (52.5)	<.001	35 (27.1)	42 (66.7)	5.37 (2.80–10.31)**
Vibration Perception Threshold *(V)*	30.37 ± 11.15	39.60 ± 10.37	<.001	34.1 ± 12.0	37.2 ± 10.8	1.31 (0.96–1.77)∧
**Muscle Strength**						
Grip Strength *(kg)*	19.84 ± 12.09	14.97 ± 8.78	.002	19.4 ± 11.2	15.3 ± 9.8	0.72 (0.52–1.00)*
**Gait**						
Gait Speed *(cm/s)*	90.57 ± 37.70	68.52 ± 29.27	<.001	85.0 ± 35.5	72.2 ± 34.4	0.68 (0.49–0.94)*
Stride Length *(cm)*	105.45 ± 33.65	85.22 ± 28.91	<.001	100.7 ± 32.4	88.5 ± 32.9	0.68 (0.49–0.93)*
CV Stride Length *(%)*	4.068 ± 3.345	5.959 ± 4.247	.001	4.46 ± 3.33	5.74 ± 4.58	1.41 (1.04–1.91)*
**Balance**						
Medio-lateral LOS *(cm)*	15.46 ± 7.48	13.24 ± 6.15	.029	15.5 ± 6.9	12.8 ± 6.7	0.67 (0.48–0.94)*
Antero-posterior LOS *(cm)*	9.72 ± 4.17	9.20 ± 4.00	.389	9.58 ± 3.99	9.05 ± 4.13	0.88 (0.64–1.20)
LOS area *(cm^2^)*	1.52 ± 1.19	1.24 ± 1.07	.091	1.46 ± 1.12	1.19 ± 1.14	0.78 (0.55–1.08)
**Cognitive**						
MMSE	26.73 ± 4.13	24.29 ± 4.33	<.001	26.3 ± 3.6	24.7 ± 5.1	0.71 (0.53–0.95)*
CDT	5.18 ± 2.10	4.50 ± 2.30	.034	5.2 ± 2.1	4.5 ± 2.5	0.75 (0.56–1.01)∧
MMSE-CDT	7.09 ± 2.72	5.48 ± 2.88	<.001	6.8 ± 2.7	5.7 ± 3.2	0.71 (0.53–0.95)*
MMSE-CDT <7	24 (25.3)	62 (62.0)	<.001	49 (37.7)	35 (55.6)	2.07 (1.12–3.81)*

Univariate Risk Factors of Experiencing at Least One Injurious Fall or Multiple (Noninjurious) Falls During 12 Months of Follow-Up (n=197).

*Notes:* Data are M ± SD or n(%). ∧*p*≤.10, *p≤.05, ** *p*≤.01.

CV  =  Coefficient of Variation; LOS  =  Limits of Stability; CI  =  Confidence Interval; MMSE  =  Mini-Mental State Examination; CDT  =  Clock Drawing Test.

Univariate analyses showed that those who suffered multiple non-injurious falls or at least one injurious fall were more likely to have diabetes mellitus, have urinary incontinence, walk with mobility aids, report falls in the previous year and report fear of falling compared to nonfallers. Fallers were also older, took significantly more medications, performed worse on hand grip strength, walked slower with smaller strides and greater variability, had smaller medio-lateral limits of stability and performed worse on the MMSE. Participants with Diabetes Mellitus performed significantly worse on all physical and cognitive measures when compared to healthy controls (Table 1).

Explanatory covariates (*p*<.1) were separately entered with diabetes into stepwise multivariate logistic regression models. The association between diabetes and falls remained significant, even after adjusting for CDT (OR = 2.13, 95%CI 1.13–4.00), age (OR = 2.08, 95%CI 1.11–3.90), MMSE (OR = 2.08, 95%CI 1.09–3.95), Vibration Perception Threshold (OR = 2.04, 95%CI 1.04–3.97), medio-lateral LOS (OR = 2.03, 95%CI 1.06–3.88) and MMSE-CDT (OR = 2.02, 95%CI 1.06–3.85). The percentage reduction of the diabetes/falls odds ratio from the logistic regression analyses was less than 10% when controlling for these covariates. Parameters that caused a substantial reduction of the diabetes/falls relationship and therefore could be considered mediators, were number of medications (20.7%, OR = 1.79, 95%CI 0.82–3.90), stride length (14.9%, OR = 1.92, 95%CI 0.99–3.72), gait velocity (14.7%, OR = 1.92, 95%CI 0.99–3.72), MMSE-CDT categorization (13.8%, OR = 1.94, 95%CI 1.00–3.78), grip strength (11.0%, OR = 2.01, 95%CI 1.06–3.79) and stride length variability (10.8%, OR = 2.01, 95%CI 1.05–3.85).

In a final stepwise logistic regression analysis all explanatory covariates (*p*≤.1) were entered together. The multivariate model identified diabetes (OR = 2.03, 95%CI 1.06–3.88) and medio-lateral LOS displacement (OR = 0.70, 95%CI 0.49–0.99) as the best predictors of future falls. Therefore, the presence of diabetes and smaller limits of stability in the medio-lateral plane are independent predictors of falls in our sample.

## Discussion

This study confirmed that diabetes mellitus is a strong predictor of falls in a mixed cohort of older adults with and without diabetes. About 41% (n=42) of the participants with diabetes were classified as fallers (35.9% experienced multiple falls and 4.9% experienced a single injurious fall). Compared to healthy controls, older adults with diabetes perform worse on physical and cognitive tests. Diabetes remained an independent risk factor of future falls, even after controlling for poor balance.

Older adults with diabetes often develop a range of long-term complications, which can explain why diabetes participants in our sample performed worse on all physical and cognitive measures. Our results confirm previous findings which commonly report more medication use, reduced peripheral nerve function, and poorer grip strength [25], gait performance [26] and balance [27] in older adults with diabetes. Similarly, the worse performance on cognitive screening measures in diabetes participants is in accordance with previous studies [28]. Older adults with diabetes also suffered more falls in the previous year and reported higher levels of fear of falling than healthy controls. We further demonstrated that older adults with diabetes were at increased risk of suffering injurious or multiple falls, even after adjusting for medical, physical and cognitive covariates.

Poor balance has previously been identified as a major risk factor for falling in older adults [7]. Accurate balance performance relies on visual, vestibular and somatosensory systems [7]. Deficiencies in these systems have proven to occur with ageing [29] and might thus lead to a loss of balance, possibly resulting in a fall. Older adults with diabetes show greater postural sways but there are many conflicting findings concerning the underlying mechanisms [30]. Our final regression model suggested that diabetes and poor balance were independently associated with falls.

In this experiment, a substantial proportion of the relationship between diabetes and future falls could be explained by more medication use, slowed walking speed and reduced cognitive performance, each of which are established risk factors for falls in older adults. First, number of medications was the strongest mediator in the diabetes/falls relationship (20.7% reduction). Previous research has consistently associated number of medications with an increased fall risk in older adults [31]. Given the multiple complications of the disease, older adults with diabetes often take a high number of medications. This was confirmed by our results; older adults with diabetes took about nine medications on average compared to four medications in older adults without diabetes. Even without medications for diabetes treatment (data not shown), the number of medications was still significantly higher for older adults with diabetes with an average of about seven medications. Second, walking performance mediated the diabetes/falls relationship in this trial and reduced the odds ratio by nearly 15 percent. Older adults with diabetes walked slower, took shorter strides and had greater stride length variability compared to controls, which confirms previous research [26]. Slowed gait can predict falls in healthy older adults [32]. Walking velocity reflects overall health and functional status and has been recommended as a potentially useful clinical indicator of well-being among older adults. The final mediator of the diabetes/falls relationship was poor cognitive performance reducing the odds ratio by 14 percent. During the past decade, researchers have provided a large body of evidence suggesting that walking performance relies on cognitive processing, executive functions and attention [33], thereby countering the former assumption of an automated human gait. Older adults with mild cognitive impairment or low cognitive reserves indeed show gait abnormalities [34], [35] and also an increased fall risk [14]. The suggested cognitive decline in patients with diabetes [36] might therefore explain why the diabetes/falls relationship is partly mediated by reduced cognitive performance. Clinicians should be aware that these factors might predispose older patients with diabetes to falling. Future research in larger samples should establish whether diabetes patients who use more medications, walk slower and show reduced cognitive performance are more prone to falling compared to diabetes patients who do not suffer from these conditions.

Current guidelines and recommendations on the management of type 2 diabetes in the general practice setting include nutrition management and increasing physical activity levels, with primary goals of controlling weight and improving metabolic control. Additionally, insulin and/or oral anti-diabetic agents are prescribed for optimizing glycemic control. Considering our finding that older adults with diabetes perform worse on physical and cognitive tests when compared to healthy controls, a comprehensive fall risk assessment, involving tests of balance, gait and cognitive functioning, should be incorporated into the clinical management of diabetes patients. Second, older adults with type 2 diabetes should be encouraged to take part in exercise programs that focus on improving balance and gait, in addition to the recommended cardiovascular fitness and resistance training. It has been shown that a challenging balance training program of adequate intensity and duration can successfully reduce fall rates in older adults [37]. Low level aerobic exercise (e.g. brisk walking for half an hour per day) is often recommended. Targeted training including gait, balance and functional strength exercises has been shown to improve gait speed, balance, muscle strength and joint mobility in patients with diabetes [38].

### Limitations

The main limitation of this study relates to a possible selection bias of the study population. Participants were recruited through advertisements or by access to patient files at the Endocrinology Clinic. Also, we acknowledge that we excluded people with severe diabetic complications that would make them unable to complete the assessments. Nevertheless, we feel that our sample does reflect the heterogeneous nature of the older adults with diabetes seen in routine practice. Also, certain potential mediators were not assessed as part of this trial. For example, poor vision has clearly been proven to adversely affect gait [39] and postural control, consequently increasing fall risk [11]. Decreased foot strength and foot pain have also independently been associated with falls [40]. Future multifactorial prospective studies should therefore include more comprehensive assessments to further enhance our understanding of the relationship between diabetes and falls.

## Conclusions

This study demonstrated that diabetes is an independent risk factor for falling, even after controlling for poor balance. Taking higher numbers of medications, poor walking performance and reduced cognitive functioning were mediators of the relationship between diabetes and faller status. These physical and cognitive measures were significantly worse in older adults with diabetes compared to older adults without diabetes. Preventive programs including systematic medication reviews, specific balance exercises and cognitive training might be beneficial in reducing fall risk in older adults suffering from diabetes.
